# Leptin Regulates Proliferation and Apoptosis in Human Prostate

**DOI:** 10.1100/2012/842301

**Published:** 2012-05-02

**Authors:** Eduardo Leze, Jorge Luiz Alves-Pereira, Sicilia Colli, Fernanda Silveira Cavalcante, Francisco José Sampaio, Cristiane da Fonte Ramos

**Affiliations:** Urogenital Research Unit, State University of Rio de Janeiro, 20551-030 Rio de Janeiro, RJ, Brazil

## Abstract

This paper aimed to evaluate the leptin role on the cellular proliferation and the expression of fibroblast growth factor 2, aromatase enzyme, and apoptotic genes in the human prostate tissue. *Methods*. Fifteen samples of hyperplasic prostate tissue were divided in four symmetric parts maintained in RPMI medium supplemented with 10% fetal bovine serum, 1 ng/mL of gentamicin, and added with 50 ng/mL leptin (L) or not (C). After 3 hours of incubation, gene expression was evaluated by real time RT-PCR. Cellular proliferation was evaluated by immunohistochemistry for PCNA. *Results*. The leptin treatment led to an increase cellular proliferation (C = 21.8 ± 0.5; L = 64.8 ± 0.9; *P* < 0.0001) and in the expression of Bax (C = 0.4 ± 0.1; L = 0.9 ± 0.2; *P* < 0.05) while Bcl-2 (C = 19.9 ± 5.6; L = 5.6 ± 1.8; *P* < 0.05), Bcl-x (C = 0.2 ± 0.06; L = 0.07 ± 0.02; *P* < 0.05), and aromatase expressions (C = 1.9 ± 0.6; L = 0.4 ± 0.1; *P* < 0.04) were significantly reduced. *Conclusion*. Leptin has an important role in maintaining the physiological growth of the prostate since it stimulates both cellular proliferation and apoptosis, with the decrement in the aromatase gene expression.

## 1. Introduction

The prostate is a sexual accessory gland that produces key substances for the efficacy of sperm to fertilize eggs within the female reproductive tract. Its secretion is a thin milky fluid that contains calcium, citrate ion, phosphate ion, a clotting enzyme, and a profibrinolysin. The slightly alkaline characteristic of the prostatic fluid may be quite important for successful fertilization of the ovum [1]. After a physiological growth to adult size, the prostate enters a maintenance phase, where prostatic cell proliferation occurs at a daily rate of 1-2% and it is counterbalanced by an equal rate of programmed cell death [2, 3].

The prostate is an androgen-dependent organ, but despite its action on the prostate, androgens can also be converted into estrogens by the enzyme aromatase [4]. The role of estrogen in the prostate is complex and particularly dependent on local signaling mechanisms to maintain a balance between the adverse and beneficial effects of this hormone [5]. The prostate is also under influence of adipokines such as leptin [6, 7]. There are six known splice variants of the leptin receptor (ObRa to ObRf), all with the same extracellular domain but with differing intracellular domains. Only the long isoform, ObRb, contains all intracellular parts able to activate the signaling pathways expressed mainly in the hypothalamus. Within the short isoforms, ObRa is the most expressed in the peripheral tissues [8]. 

The benign prostate hyperplasia (BPH) is the disease in which the prostate shows an abnormal growth. The prevalence of histologic marks of BPH is age related, rising up to 88% in the ninth decade of life [9]. Nonetheless, the etiology of BPH is far from being completely understood, despite the great efforts showed by molecular and clinical studies. Most of these studies are focused on hormonal factors, postulating that advanced age and the presence of normal levels of serum testosterone were the two best-established conditions for the development of BPH. Based on the role played in several models of human pathology, cellular proliferation and apoptosis have been investigated extensively in the last decade, achieving significant improvements in our knowledge of both physiologic prostate growth and BPH pathogenesis.

Leptin, the most well-characterized adipokine involved in reproduction, is known by its effect in decreasing caloric intake and increasing energy expenditure [10]. Besides follicle stimulating and luteinizing hormones, leptin has an important role regulating the reproductive system [10]. It is also known that there is a link between obesity and BPH [11]. Based on these facts, the aim of the present study was to evaluate, using human hyperplasic prostate tissue, how leptin can regulate proliferation and the expression of fibroblast growth factor 2 (FGF2), aromatase enzyme, and apoptotic genes.

## 2. Material and Methods

This project was approved by the ethical committee of the Biology Institute of the State University of Rio de Janeiro. Hyperplasic prostate tissue samples obtained by transvesical prostatectomy in fifteen patients with enlarged prostates that caused mainly postvoiding residual urine and several LUT's symptoms were used. Patients presenting one or more of the conditions below were excluded from the study: medication use for LUT'S, abnormal glucose fasting, BMI above 25, and recent urinary instrumentation. Those measures were used to avoid bias, such as apoptosis induction, interactions between insulin and leptin, inflammatory condition with leptin increase in obese individuals, and urinary tract infection, respectively. Other disturbances if under control were accepted. 

A transversal piece of each adenoma was taken under sterile conditions, then immersed into the culture medium, at 4°C and sent to the lab immediately. Each sample was divided in four symmetric parts which were maintained in RPMI (Roswell Park Memorial Institute) medium supplemented with 10% fetal bovine serum and 1 ng/mL of gentamicin. After one hour of incubation, the medium was changed by the same medium as above supplemented (L) or not (C) with 50 ng/mL leptin. After 3 hours of incubation, RNA was extracted using Trizol reagent (Invitrogen, Carlsbad, CA) according to the manufacturer's protocol from one tissue piece of each group. Then 1 *μ*g RNA sample was used in a 20 *μ*L cDNA reaction using oligo-dT and the superscript III cDNA synthesis system (Invitrogen, Carlsbad, CA) according to the manufacturer's protocol. The gene expression of FGF2, aromatase, Bax, Bcl-x, and Bcl-2 was evaluated by real time polymerase chain reaction (primer sequences listed in Table 1). The sample was also submitted to histopathology analysis to confirm it as benign hyperplasic tissue. The sequence of primers is listed on Table 1.

Cellular proliferation was evaluated by immunohistochemistry in the other tissue sample of each group. Five deparaffiniled tissue sample sections in each group were hydrated, treated with buffer TRIS-EDTA (pH 9.0) overnight at 60°C for antigen retrieval, and then treated with 3% hydrogen peroxide solution in methanol for 10 min to block endogenous peroxidase activity. These steps were followed by washing the sections in PBS and subsequently incubating for 10 min at room temperature with 10% goat serum to block unspecific binding. The sections were then incubated for 2 h at 37°C with mouse anti-PCNA (Proliferating Cell Nuclear Antigen; Invitrogen, CA, USA, cat#180110), diluted 1 : 200 in PBS with 1% BSA. Sections were then washed in PBS and incubated at room temperature for 20 min with biotinylated secondary antibody followed by incubation at room temperature for 10 min with streptavidinperoxidase conjugate (Histostain-Plus Kit, Invitrogen, CA, USA). Sections were washed in PBS, then revealed with liquid diaminobenzidine (Histostain-Plus Kit, Invitrogen, CA, USA), and then counterstained with hematoxylin. The negative controls were processed by replacing the primary antibody with PBS and no indication of staining was observed. Images were digitized using an Olympus DP70 (12.5 megapixels, Tokyo, Japan) video camera coupled to a BX51 Olympus light microscope (Tokyo, Japan).

The histomorphometric evaluation of cellular proliferation was performed in a final increase of 600x and estimated by the number of stained nuclei of epithelial and stromal cells counted in a area of 0.04 mm^2^ with a specific software (Image_J 1.41; NIH, Bethesda, USA).

Data were reported as mean ± SEM. Statistical significance of experimental observations was determined by Student's t test. The level of significance was set at *P* ≤ 0.05.

## 3. Results

**Figure 1:**
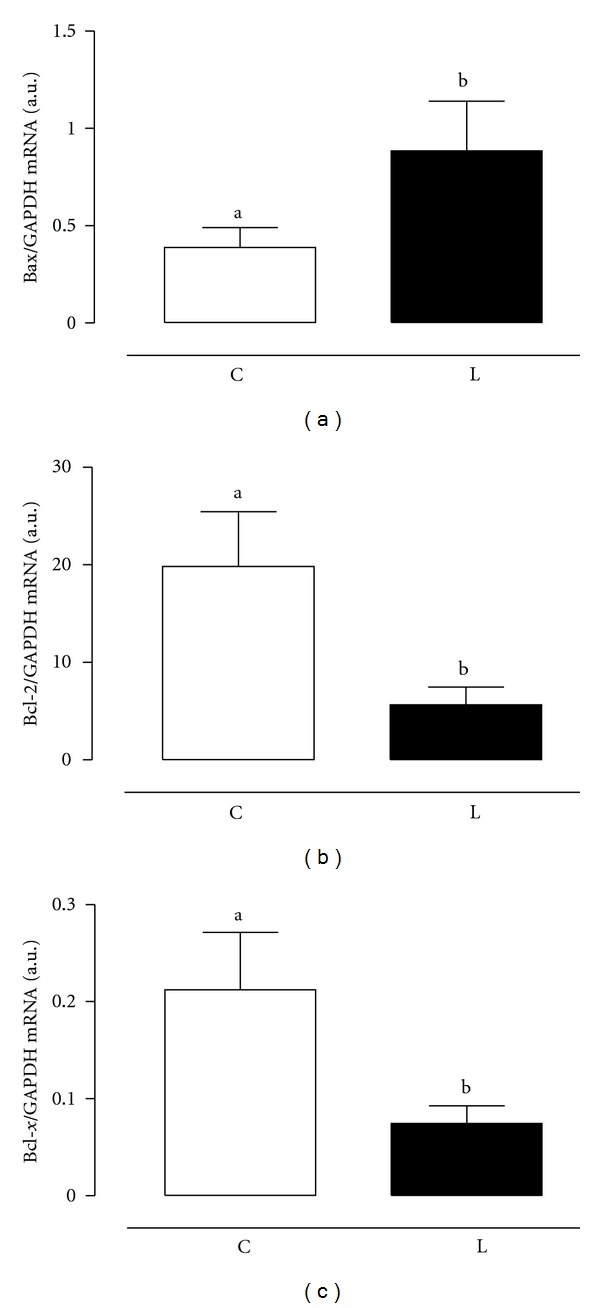
Gene expression of Bax (a), Bcl-2 (b), and Bcl-x (c) in the human hyperplasic prostate tissue after leptin treatment, 50 ng/mL, (Leptin) or not (Control) for 3 hours. GAPDH was used as an internal control. Primer sequences are listed in the Table 1. Data are represented as mean ± SEM of 15 individuals. Different letters mean statistically significance.

Figure 1 shows the apoptotic gene expression results evaluated by real-time PCR. The leptin treatment leads to a significant (*P* < 0.05) increase in the expression of the pro apoptotic gene Bax while the antiapoptotic genes Bcl-2 and Bcl-x expressions were significantly (*P* < 0.05) reduced. Comparing the expression of both antiapoptotic genes, Bcl-2 was more expressed in both groups than Bcl-x. The difference was about 20 times more in C group and 10 times more in L group. 

**Figure 2:**
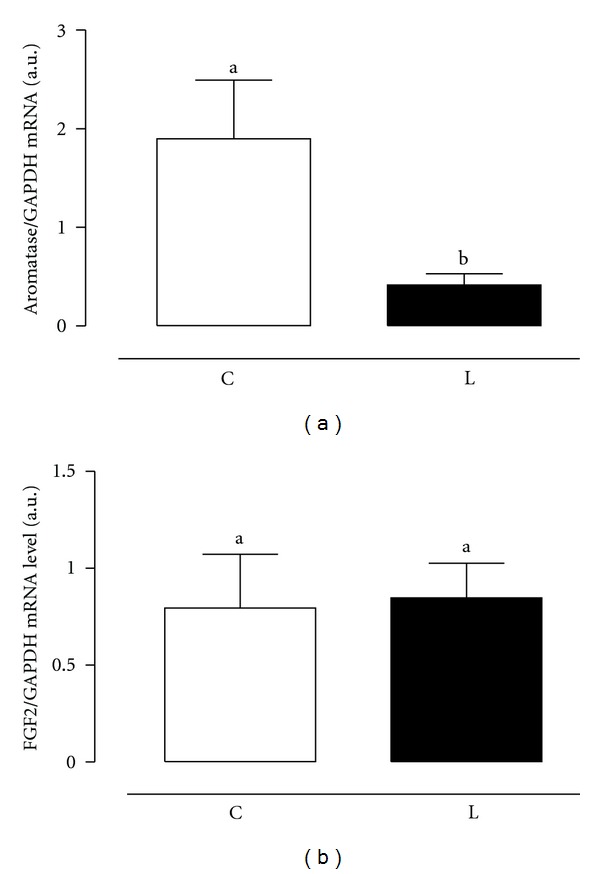
Gene expression of FGF2 (a) and aromatase (b) in the human hyperplasic prostate tissue after leptin treatment, 50 ng/mL, (Leptin) or not (Control) for 3 hours. GAPDH was used as an internal control. Primer sequences are listed in the Table 1. Data are represented as mean ± SEM of 15 individuals. Different letters mean statistically significance.

The expression of FGF2 and aromatase genes is shown in Figure 2. While there was no significant alteration in the FGF2 expression, the aromatase expression was significantly (*P* < 0.04) reduced by the leptin treatment.

**Figure 3:**
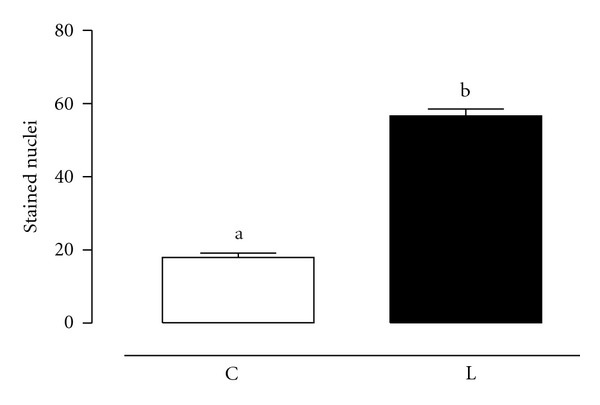
Cellular proliferation evaluated by PCNA technique through immunohistochemistry, in the human hyperplasic prostate tissue after leptin treatment, 50 ng/mL, (Leptin) or not (Control) for 3 hours. Data are represented as mean ± SEM of 15 individuals. Different letters mean statistically significance.

**Figure 4:**
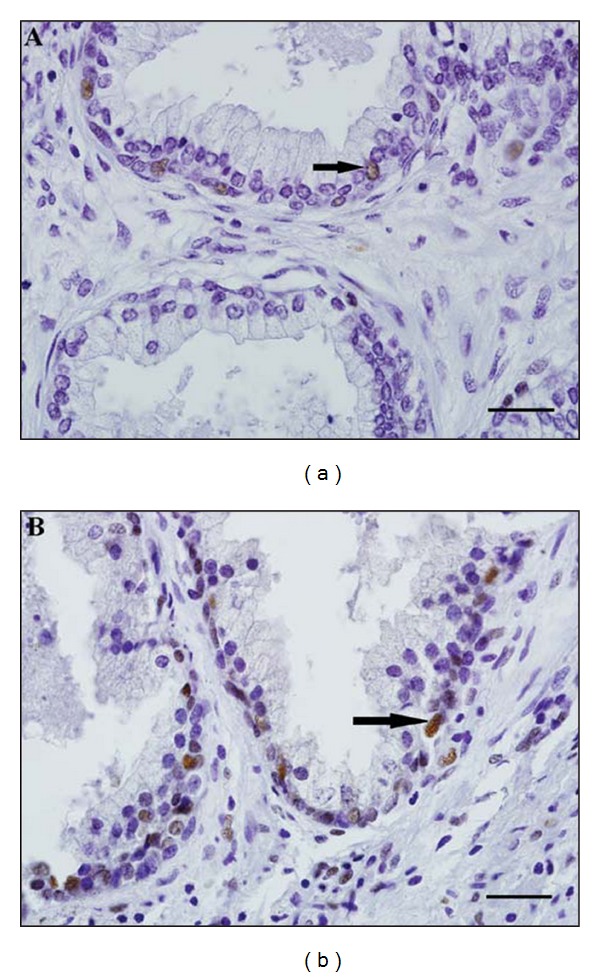
Photomicrography showing immunohistochemistry of PCNA, in the human hyperplasic prostate tissue after leptin treatment, 50 ng/mL, (Leptin) or not (Control) for 3 hours. Magnification of 600x.

 The cellular proliferation was evaluated by PCNA immunohistochemistry. The leptin treatment resulted in a significant (*P* < 0.0001) increase in the number of stained cells (Figure 3). The immunohistochemistry photomicrograph is shown in Figure 4.

## 4. Discussion

Leptin is one of the most important factors, besides follicle stimulating and luteinizing hormones, in the regulation of reproductive system [10]. As in other tissues of the reproductive system, the prostate not only expresses leptin receptors but also synthesizes the hormone itself [12, 13]. Leptin has been associated with prostate diseases but the real role of this hormone in the normal or abnormal prostate tissue has not yet been clarified.

Several papers show that there is a link between obesity and prostate neoplasms, such as BPH and prostate cancer [14, 15]. An increase in leptin expression in prostate tissue is also related to the progression and the degree of malignancy of PCa [16]. It has been reported that the blood concentration of leptin, as well as the amount of leptin in the tissue, is related to the progression of several cancers besides the prostate [17, 18]. It is also known that leptin stimulates cellular proliferation of benign prostatic hyperplasia. In spite of those papers, we have no knowledge about the direct effects of leptin in the human prostate gland. So, we believe that this is the first time a direct effect of leptin has been shown in the human prostate using an in vitro culture tissue assay.

The equilibrium between proliferation and apoptosis is very important to maintain the physiological growth of the adult prostate [2, 3]. Based on this statement, any factor that alters that equilibrium could result in an abnormal growth of the prostate. The fact that leptin treatment caused an increase in the expression of the proapoptotic gene Bax and a reduction in the expression of both antiapoptotic genes Bcl-2 and Bcl-x suggests that one of the leptin functions in the prostate is to favor the apoptosis reaction. Corroborating the literature [19], our results also show that Bcl-2 is more expressed than the other apoptotic genes. Leptin treatment did not change that relation.

PCNA immunohistochemistry results showed that leptin-stimulated proliferation in the prostate corroborating a previous study that showed that leptin could stimulate proliferation and migration in prostate cell lines [20]. The combination of these results suggests that in the prostate, leptin is important to maintain the normal physiological adult growth balance by favoring both apoptosis and proliferation reactions. These results are in agreement with the leptin role in human ovarian cells where this hormone has the ability to stimulate both proliferation and apoptosis [21].

The prostate is known to be strongly influenced by androgens whose effects are thought to be mediated by interactions between epithelial and stromal compartments, through a complex network of paracrine and autocrine factors. Basic fibroblast growth factor was the first growth factor to be isolated in the prostate with mitogenic effect on epithelial or stromal cells [22]. Diidrotestosterone is the most potent androgen affecting the prostate. It has an important role on prostate growth modulation, since it influences the prostatic homeostasis, which is controlled by mitogenic and inhibitor factors, responsible for cellular proliferation and cell death, respectively [23]. As there was no change in the FGF2 gene expression by the leptin treatment, we could hypothesize that this hormone has a local and direct effect upon proliferation that is not secondary to FGF.

Estrogen regulation has also been considered as one of the hormonal risk factors in association with the development of benign prostatic hyperplasia and prostate cancer [24–26]. An increase in aromatase expression is shown in several types of cancer including prostate [27] and breast cancer [28]. Leptin has been shown to increase aromatase expression and activity in human-luteinized granulosa [29] and MCF-7 cell line [30]. In this study, we observed that leptin treatment led to a decrease in aromatase expression suggesting that in the prostate leptin could have a protective effect against abnormal growth, especially considering the proliferation and apoptosis effects of the hormone.

It has been shown in a mouse model that the prostate gland is dependent upon both androgenic and estrogenic responses and none of these hormones alone is sufficient to evoke aberrant patterns of growth, resulting in malignancy [31]. So, despite the protective effect that leptin seems to have in this in vitro model, we cannot forget that, in vivo, leptin actions in the prostate gland probably are dependent on other hormones. So, the lack of synergism among leptin and others hormones in the in vitro system could be responsible for the difference between in vivo and in vitro effects of the hormone.

## 5. Conclusion

We can conclude that in the human prostate leptin alone seems to have an important role in maintaining the normal physiological growth of the organ. This effect could be reached by the fact that leptin stimulates both cellular proliferation and apoptosis along with the decrement in the aromatase gene expression.

## Figures and Tables

**Table 1:** Sequence of primers.

Gene	Sequence
Aromatase	Forward primer gcttctcatcgcagagtatccgg Reverse primer caagggtaaattcattgggcttgg
Bax	Forward primer ggggacgaactggacagtaa Reverse primer cagttgaagttgccgtcaga
Bcl-2	Forward primer atgtgtgtggagagcgtcaa reverse primer acagttccacaaaggcatcc
Bcl-x	Forward primer cgtagacaaggagatgcaggt Reverse primer accagcggttgaagcgctcct
FGF-2	Forward primer ccgttacctggctatgaagg Reverse primer actgcccagttcgtttcagt
GAPDH	Forward primer tcgtggaaggactcatgac Reverse primer ccatcacgccacagttt
